# Supplementary figures and images for: Development of a noninvasive photograph-based method for the evaluation of body condition in free-ranging brown bears
Source: PeerJ. 2020 Sep 18;8:e9982. doi: 10.7717/peerj.9982 (PMC7505064; doi:10.7717/peerj.9982)

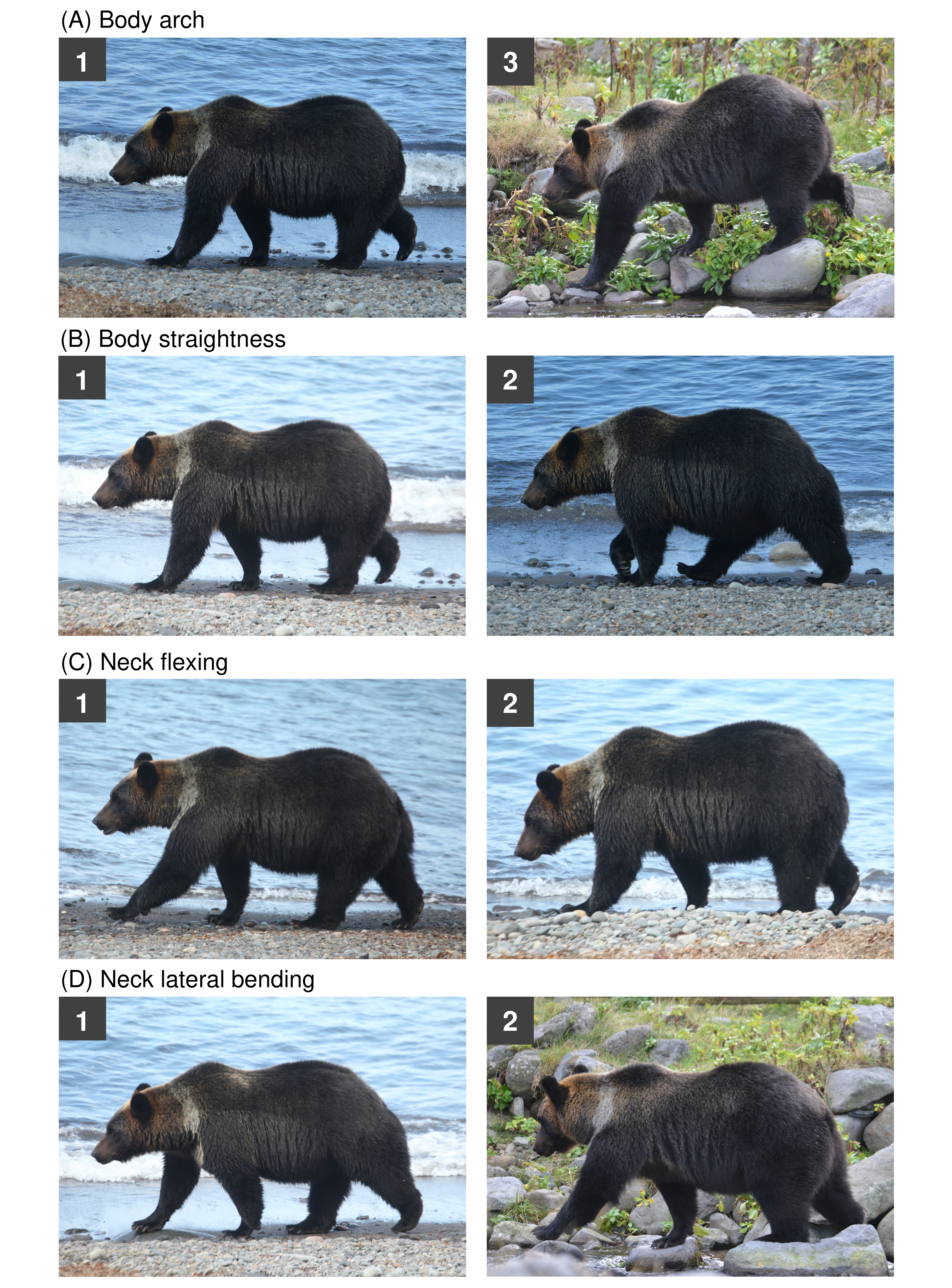

Supplement: Figure S1 — (A) body arch, (B) body straightness, (C) neck flexing, and (D) neck lateral bending. See Table S1 for definition of the different grades for each attribute. Photo credit: Yuri Shirane. [file peerj-08-9982-s001.png]

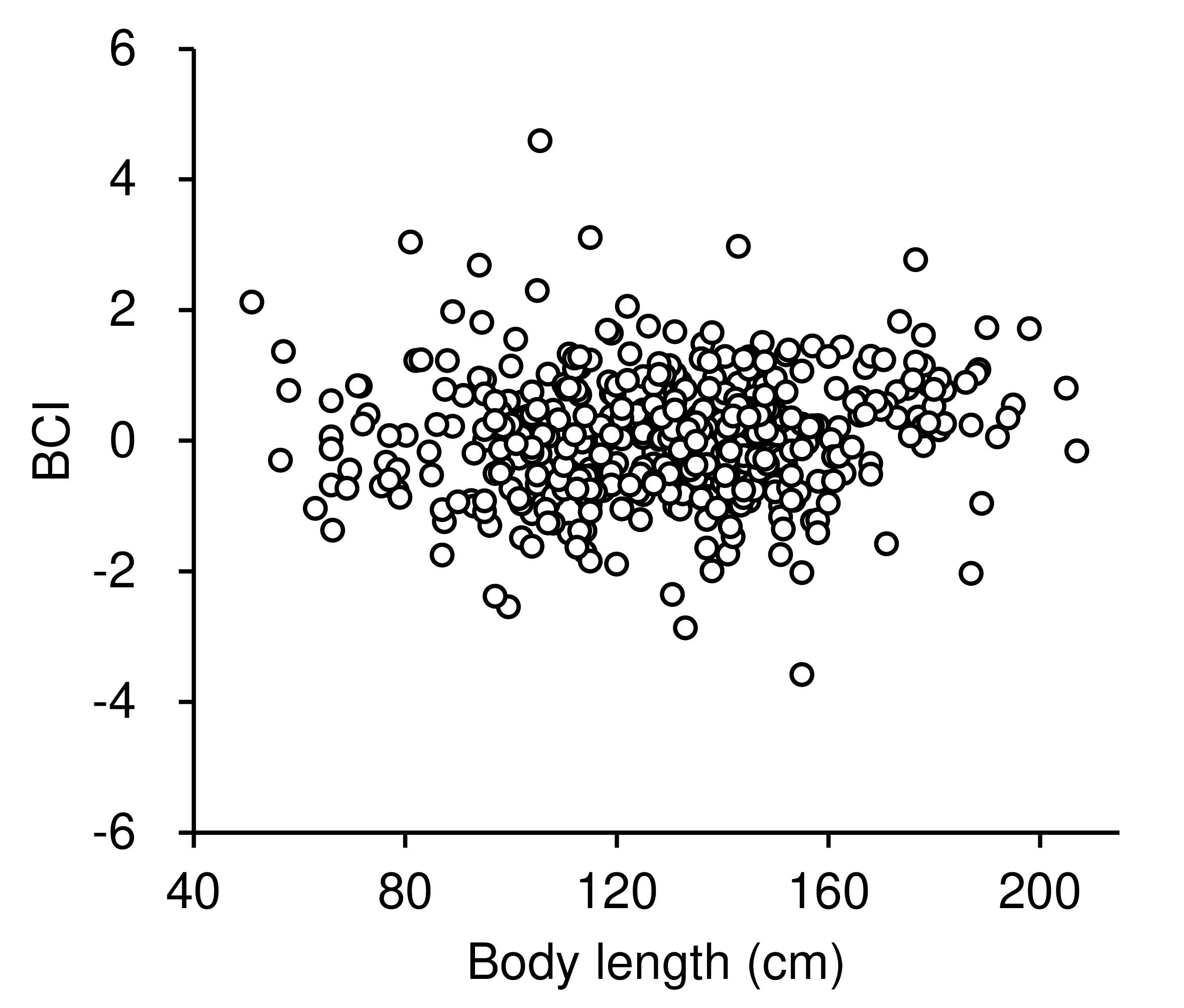

Supplement: Figure S2 — Pearson’s correlation values were r = 0.037 (p = 0.39). [file peerj-08-9982-s002.jpg]

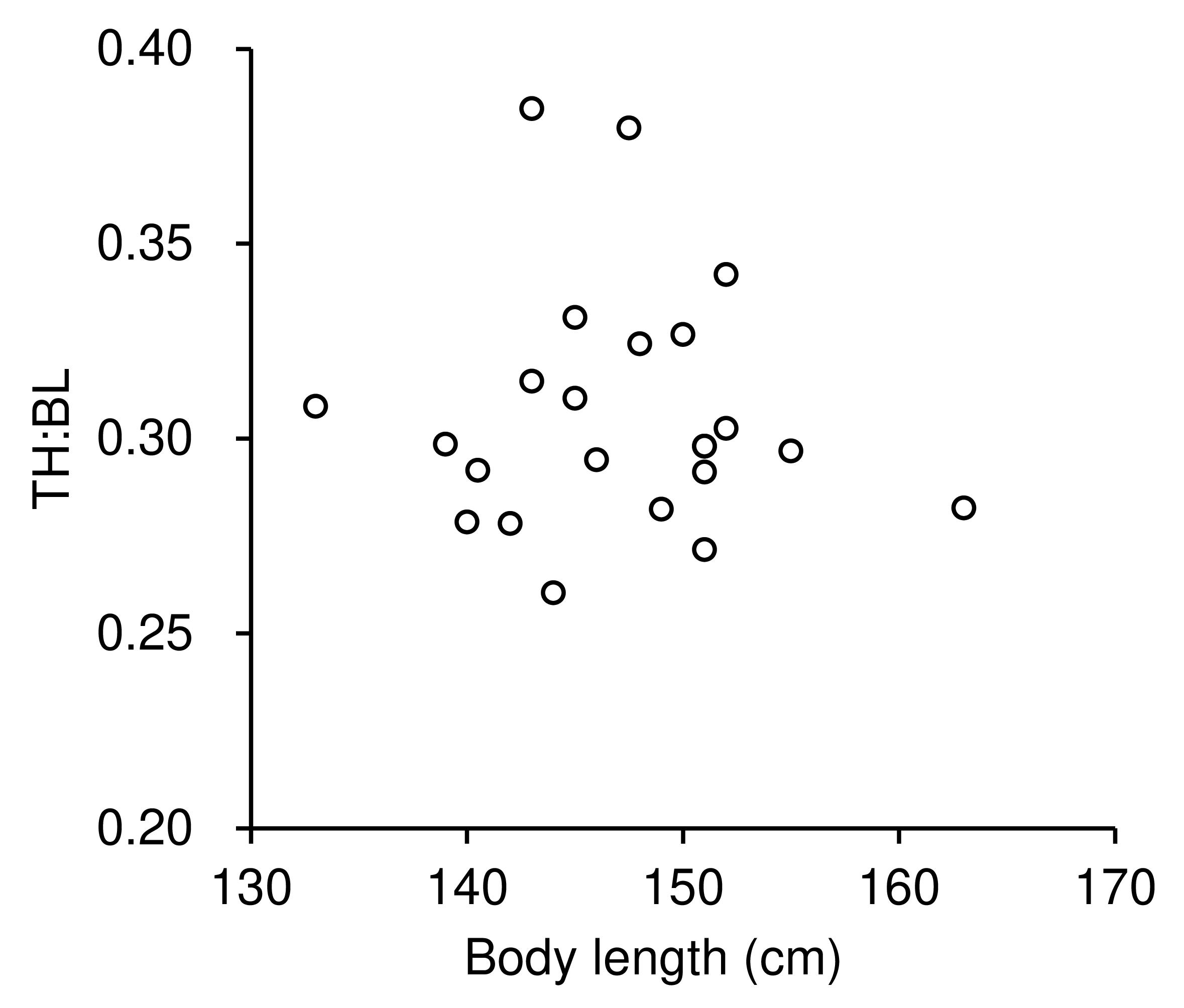

Supplement: Figure S3 — Pearson’s correlation values were r = –0.068 (p = 0.73). [file peerj-08-9982-s003.jpg]
