# Supplementary material for: Development of a noninvasive photograph-based method for the evaluation of body condition in free-ranging brown bears
Source: PeerJ. 2020 Sep 18;8:e9982. doi: 10.7717/peerj.9982 (PMC7505064; doi:10.7717/peerj.9982)
Supplement: Table S1 — See Fig. S1 for example pictures of each grade for each attribute of bear posture. [file peerj-08-9982-s004.pdf]

Supplemental Information

PeerJ

Development of a noninvasive photograph-based method for the evaluation of body condition in free-ranging brown bears

Yuri Shirane[1], Fumihiko Mori[1], Masami Yamanaka[2], Masanao Nakanishi[2], Tsuyoshi Ishinazaka[2], Tsutomu Mano[3], Mina Jimbo[1], Mariko Sashika[1], Toshio Tsubota[1], and Michito Shimozuru[1]

[1] Laboratory of Wildlife Biology and Medicine, Graduate School of Veterinary Medicine, Hokkaido University, Sapporo, Hokkaido, Japan

[2] Shiretoko Nature Foundation, Shari, Hokkaido, Japan

[3] Hokkaido Research Organization, Sapporo, Hokkaido, Japan

Corresponding Author:

Michito Shimozuru

Kita 18 Nishi 9, Kita-ku, Sapporo, Hokkaido, 060-0818, Japan

Email address: shimozuru@vetmed.hokudai.ac.jp

Table S1:

Definition of grades (scores) used for each photograph attribute to select photographs for assessing body condition.

See Fig. S1 for example pictures of each grade for each attribute of bear posture.

Attribute	Score 1 (good)	Score 2 (medium)	Score 3 (poor)
**Photographing condition**			
(A) Camera focus	The picture is sharp with the contour of the bear's body clearly visible.	The picture is blurry, but still clear enough to make out the contour of the bear's body.	The picture is too blurry to make out the contour of the bear's body.
(B) Camera tilt	The dorsal and ventral suface of the bear are vertical to the camera.	The dorsal and ventral suface of the bear deviate slightly from the vertical with the camera.	The dorsal and ventral suface of the bear deviates significantly from the vertical with the camera.
(C) Body angle	The body axis of the bear is perpendicular to the camera.	The body axis of the bear is angled slightly in the parallel plane, either back or forth.	The body axis of the bear is angled significantly in the parallel plane, either back or forth.
(D) Torso height measurability	Both the highest part of the waist and the lowest part of the abdomen are clearly visible.	The highest part of the waist or the lowest part of the abdomen is unclear or partly obscured, but can still be approximated.	The highest part of the waist and/or the lowest part of the abdomen are not visible due to another animal or object.
(E) Body/torso length measurability	The tip of nose (for HBL, EBL and PBL), the highest part of the shoulder (for PBL and HTL), and the base of tail are clearly visible.	The tip of nose , the highest part of the shoulder, or the base of tail is unclear or partly obscured, but can still be approximated.	The tip of nose , the highest part of the shoulder, and/or the base of tail are not visible due to another animal or object.
**Bear posture**			
(A) Body arch	No visible arching of the body. The bear stands parallel to the ground.	-	The legs or the central part of the body is significantly lifted or dropped.
(B) Body straightness	Right/left forelimb and hindlimb are in front of the other. The hip joint is not fully extended.	Right/left forelimb and hindlimb are inside the body or either of the legs is fully extended.	-
(C) Neck flexion	The angle between the ground surface and the line connecting the base of tail and the tip of nose is < 13 degree.	The angle between the ground surface and the line connecting the base of tail and the tip of nose is ≥ 13 degree.	-
(D) Neck lateral bending	The neck of the bear is not bending laterally. The left and right ears are aligned.	The neck of the bear is bending to the side. The left and right ears do not overlap.	-
