# Supplementary material for: Development of a noninvasive photograph-based method for the evaluation of body condition in free-ranging brown bears
Source: PeerJ. 2020 Sep 18;8:e9982. doi: 10.7717/peerj.9982 (PMC7505064; doi:10.7717/peerj.9982)
Supplement: Table S2 — Body condition index (BCI) was calculated from their actual measurements and the ratio of torso height to horizontal torso length (TH:HTL) was calculated from their photographs. [file peerj-08-9982-s005.pdf]

Supplemental Information

*PeerJ*

# Development of a noninvasive photograph-based method for the evaluation of body condition in free-ranging brown bears

Yuri Shirane[1], Fumihiko Mori[1], Masami Yamanaka[2], Masanao Nakanishi[2], Tsuyoshi Ishinazaka[2], Tsutomu Mano[3], Mina Jimbo[1], Mariko Sashika[1], Toshio Tsubota[1], and Michito Shimozuru[1]

[1] Laboratory of Wildlife Biology and Medicine, Graduate School of Veterinary Medicine, Hokkaido University, Sapporo, Hokkaido, Japan

[2] Shiretoko Nature Foundation, Shari, Hokkaido, Japan

[3] Hokkaido Research Organization, Sapporo, Hokkaido, Japan

Corresponding Author:

Michito Shimozuru

Kita 18 Nishi 9, Kita-ku, Sapporo, Hokkaido, 060-0818, Japan

Email address: shimozuru@vetmed.hokudai.ac.jp

**Table S2:**

**The capture date and photographing period for seven adult female brown bears captured in the Rusha area of the Shiretoko Peninsula, Hokkaido, 2014–2016.**

Body condition index (BCI) was calculated from their actual measurements and the ratio of torso height to horizontal torso length (TH:HTL) was calculated from their photographs.

| | | Capturing | | Photographing | | |
|---|---|---|---|---|---|---|
| Bear-ID | Year | Date | BCI | Date | TH:HTL | *n* |
| BE | 2015 | 4 July | -0.395 | 3–4 July | 0.651 | 4 |
| DR | 2016 | 6 August | 0.243 | 6 August | 0.670 | 2 |
| GI | 2015 | 29 September | -0.992 | 29 September | 0.602 | 4 |
| KR | 2014 | 25 August | 0.252 | 25–28 August | 0.651 | 2 |
| LI | 2016 | 5 July | -0.378 | 3–5 July | 0.662 | 4 |
| RI | 2016 | 6 August | -0.499 | 6–7 August | 0.625 | 4 |
| WK | 2016 | 4 July | 0.135 | 1–4 July | 0.647 | 4 |
